# Construction of Methotrexate-Loaded Bi_2_S_3_ Coated with Fe/Mn-Bimetallic Doped ZIF-8 Nanocomposites for Cancer Treatment Through the Synergistic Effects of Photothermal/Chemodynamic/Chemotherapy

**DOI:** 10.1021/acsami.4c13465

**Published:** 2024-10-17

**Authors:** Pranjyan Dash, Nandini Nataraj, Pradeep Kumar Panda, Ching-Li Tseng, Yu-Chien Lin, Rajalakshmi Sakthivel, Ren-Jei Chung

**Affiliations:** 1 Department of Chemical Engineering and Biotechnology, National Taipei University of Technology (Taipei Tech), Taipei 10608, Taiwan; 2 Department of Chemical Engineering and Materials Science, Yuan Ze University, Taoyuan City 32003, Taiwan; 3 Graduate Institute of Biomedical Materials and Tissue Engineering, College of Biomedical Engineering, Taipei Medical University, Taipei City 110, Taiwan; 4 International Ph. D. Program in Biomedical Engineering, College of Biomedical Engineering, Taipei Medical University, Taipei city 110, Taiwan; 5 Research Center of Biomedical Device, College of Biomedical Engineering, Taipei Medical University, Taipei city 110, Taiwan; 6 International Ph. D. Program in Cell Therapy and Regenerative Medicine, College of Medicine, Taipei Medical University, Taipei city 110, Taiwan; 7 School of Materials Science and Engineering, Nanyang Technological University, 50 Nanyang Avenue, Singapore 639798, Singapore; 8 ZhongSun Co., LTD, New Taipei City 220031, Taiwan; 9 High-value Biomaterials Research and Commercialization Center, National Taipei University of Technology (Taipei Tech), Taipei 10608, Taiwan

**Keywords:** metal-doped ZIF-8, metal−organic frameworks, Bi_2_S_3_ nanorods, chemodynamic therapy, photothermal therapy, chemotherapy

## Abstract

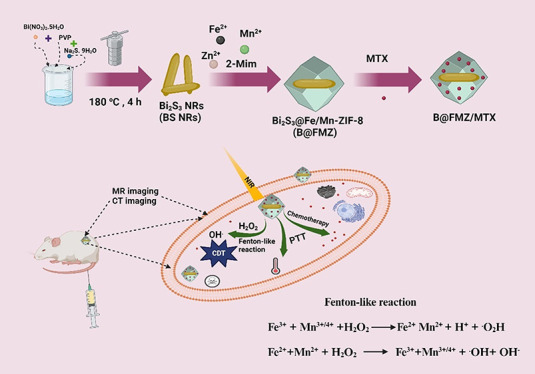

A combination of therapeutic modalities in a single nanostructure is crucial for a successful cancer treatment. Synergistic photothermal therapy (PTT) can enhance the effects of chemodynamic therapy (CDT) and chemotherapy, which could intensify the therapeutic efficacy to induce cancer cell apoptosis. In this study, Fe and Mn on a zeolitic imidazolate framework (ZIF-8) (Fe/Mn-ZIF-8; FMZ) were synthesized through ion deposition. Furthermore, bismuth sulfide nanorods (Bi_2_S_3_ NRs; BS NRs) were synthesized via a hydrothermal process and coated onto FMZ to generate the core–shell structure of the Bi_2_S_3_@FMZ nanoparticles (B@FMZ). Next, methotrexate (MTX) was loaded effectively onto the porous surface of ZIF-8 to form the B@FMZ/MTX nanoparticles. The Fenton-like reaction catalyzes Fe^2+^/Mn^2+^ ions by decomposing H_2_O_2_ in the tumor microenvironment, resulting in the formation of toxic hydroxyl radicals (·OH), which promotes the CDT effect of killing cancer cells. Furthermore, under 808 nm laser irradiation, these B@FMZ nanoparticles showed a strong PTT effect, owing to the presence of intense BS NRs as a photothermal agent. The B@FMZ nanoparticles exhibited a prominent drug release efficiency of 87.25% at pH 5.5 under near-infrared laser irradiation due to the PTT effect can promote the drug delivery performance. The B@FMZ nanoparticles were subjected to dual-modal imaging, guided magnetic resonance imaging, and X-ray computed tomography imaging. Both *in vitro* and *in vivo* results suggested that the B@FMZ/MTX nanoparticles exhibited enhanced antitumor effects through the combined therapeutic effects of PTT, CDT, and chemotherapy. Therefore, these nanoparticles exhibit good biocompatibility and are promising candidates for cancer treatment.

## Introduction

Chemodynamic therapy (CDT) is considered a promising strategy for cancer therapy owing to its noninvasiveness and enhanced selectivity. CDT can catalyze metal ions (Fe^2+^, Cu^2+^, and Mn^2+^) to produce highly toxic hydroxyl radicals (·OH) by reacting with overexpressed hydrogen peroxide (H_2_O_2_) in the tumor microenvironment (TME).^1−6^ This phenomenon occurs via Fenton or Fenton-like reactions that can induce apoptosis in cancer cells. However, combined therapeutic strategies have great potential for effectively enhancing the therapeutic effects of cancer treatment. Photothermal therapy (PTT) induces tumor ablation through the conversion of near-infrared (NIR) light energy into hyperthermia at the site of the tumor.^1,7,8^ PTT has attracted considerable interest in cancer therapy owing to its high photothermal stability, spatial and temporal resolution, enhanced conversion efficiency, minimal invasiveness, high selectivity, good biocompatibility, and nontoxicity.^9−14^ Chemotherapy is an efficient strategy for cancer treatment. However, chemotherapy is associated with serious side effects and also a lack of tumor-specific drug resistance.^15,16^ PTT-guided treatment improves chemotherapeutic efficiency and enhances targeted drug delivery to the tumor microenvironmment.^17^ Moreover, the synergistic therapeutic effects of PTT/CDT/chemotherapy have enormous potential in cancer treatment to improve therapeutic efficiency and overcome complications.^18^

Metal–organic frameworks (MOFs) have received significant attention in regard to oncology treatment because of their high porosity, good biodegradability, high surface area, reliable size, excellent biocompatibility, and enhanced functionality.^1,2,19−25^ Moreover, MOF-based materials can be used as nanocarriers for cancer therapy. Zeolite imidazole framework-8 (ZIF-8) is an effective nanocarrier owing to its suitable fabrication, pH responsiveness, and prominent drug-loading efficiency.^26−30^ Therefore, metal-catalyzed MOF-based materials can trigger Fenton-like reactions via the CDT effect in order to generate toxic ·OH radicals that can kill cancer cells. ZIF-8 has been widely used in nanomedicine as a pH-dependent, degradable drug carrier owing to its highly porous properties. ZIF-8 exhibits sluggish photocatalytic activity owing to its higher bandgap. Various single-metal doping strategies have been developed to overcome these limitations. Cu-doped ZIF-8 performed a 2.61 eV bandgap, as reported by Li et al.^31^ Fe-doped ZIF-8-based materials possess a band gap of 2.2 eV that can be utilized for intense magnetic behavior via the CDT effect. Bimetal doping effects based on ZIF-8, including Mn and Fe, can induce narrow band gaps, enhance the CDT effect through the Fenton reaction, and excite longer visible light regions. Furthermore, Mn-doped MOF enables intense drug release in acidic tumor microenvironments. Therefore, bimetal-doped MOF materials are excellent drug carrier ·OH generation through endogenous H_2_O_2_ degradation leads to the CDT effect and excitation by visible long wavelength light. Transition metal ions with high redox valences can be attributed to the MOF material’s glutathione-responsive synthesis process. Synergistic therapeutic efficacy can improve the CDT effect via the Fenton reaction involving the decomposition of H_2_O_2_ using a Fenton-based catalyst. Drug loading with bimetal-doped ZIF-8 materials can enhance chemotherapeutic and chemodynamic therapies. Bismuth sulfide (Bi_2_S_3_) is an n-type semiconductor with a bandgap of 1.3–1.7 eV. Bi_2_S_3_-based nanomaterials possess good biocompatibility, photothermal properties, intense photothermal conversion efficiency, good biodegradability, prolonged blood circulation, enhanced X-ray computed tomography (CT) contrast agents and photoacoustic (PA) imaging, nontoxicity, low cost, and strong NIR absorption efficiency.^32−39^ Bi is a high-Z element that exhibits a high X-ray attenuation coefficient and considered as a promising CT contrast agent.^40^ It can absorb strong NIR light, which can liberate and enhance the photothermal properties. Thus, Bi_2_S_3_-based materials can be utilized for intense CT imaging-guided PTT effect through irradiation with NIR light. Therefore, a combination of Bi_2_S_3_ and ZIF-based metal-doped materials is a promising candidate for cancer theranostic treatment under NIR irradiation. Hence, these synergistic samples can be enhanced by ·OH radicals through the Fenton reaction via H_2_O_2_ decomposition. Furthermore, combined semiconductor heterostructured nanomaterials can promote the synergistic therapeutic effects of PTT/CDT/chemotherapy, which can induce cell apoptosis and intensify tumor growth inhibition with NIR light irradiation for cancer treatment. These semiconductor heterojunction materials exhibit strong absorption in the NIR region, superior electron–hole pair separation, and improved photocatalytic activity. However, these combined materials have rarely been investigated for theranostic cancer treatment.

**Scheme 1:**
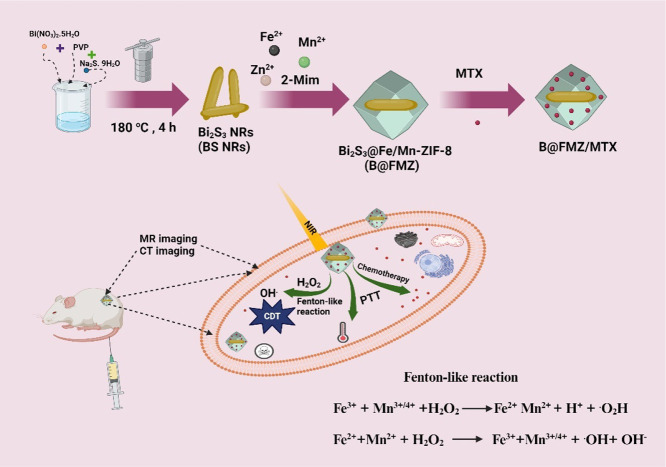
Schematic Diagram of B@FMZ/MTX Nanoparticles through the Multimodal Imaging-Guided Synergistic Therapeutic Effects of CDT, PTT, and Chemotherapy for Cancer Theranostics

Herein, we have designed bimetal-doped, that is, Fe and Mn, on ZIF-8 (Fe/Mn-ZIF-8; FMZ) through the ion deposition method. Furthermore, bismuth sulfide nanorods (Bi_2_S_3_ NRs; BS NRs) were synthesized via a hydrothermal process and coated on FMZ to produce the core@shell structure of Bi_2_S_3_@FMZ nanoparticles (B@FMZ). Next, methotrexate (MTX) was loaded effectively onto the porous surface of ZIF-8 to form B@FMZ/MTX nanoparticles. The Fenton activity of Fe/Mn-ZIF-8 can degrade H_2_O_2_ to produce intense ·OH radicals that result in the CDT effect. BS NRs can exhibit an enhanced PTT effect under NIR laser irradiation and can be utilized as an intense CT contrast agent to induce cancer cell apoptosis. Moreover, PTT enhances the efficacy of CDT and chemotherapy. Therefore, the B@FMZ/MTX nanoparticles possessed intense chemotherapeutic activity, a high photothermal therapy effect, and an improved CDT effect. Furthermore, these nanoparticles can be utilized in intense magnetic resonance imaging (MRI) and CT imaging for cancer treatment. The biocompatibility, cytotoxicity, and antitumor efficacy of nanoparticles have been extensively discussed through both *in vitro* and *in vivo* investigations. The synthesized nanoparticles can be considered promising candidates for cancer treatment through the synergistic therapeutic effects of multimodal imaging-guided MRI/CT imaging and CDT/PTT/chemotherapy with NIR laser irradiation. Scheme 1 presents a schematic depiction of the B@FMZ/MTX nanoparticles, which exhibit multimodal imaging-guided synergistic therapeutic effects for cancer treatment via PTT, CDT, and chemotherapy.

## Experimental Section

The materials and characterizations are provided in the Supporting Information.

### Synthesis of BS NRs

A hydrothermal process was employed to synthesize the BS NRs. First, Bi(NO_3_)_3_·5H_2_O (0.45 g) was suspended into ethylene glycol (60 mL) and stirred for 0.5 h, followed by adding PVP (0.5 g) onto the above solution and stirred another 30 min. Next, the Na_2_S·9H_2_O (0.2 g/mL) was added to this solution, incubated for 15 min, and stirred in order to obtain a homogeneous mixture. The homogeneous solution was transferred into the autoclave, heated (180 °C, 4 h), and cool down at ambient temperature. The precipitate obtained was centrifuged and rinsed several times with DI water and ethanol. Finally, the product was dried in a vacuum oven.

### Synthesis of ZIF-8 and FMZ Nanoparticles

To synthesize B@FMZ nanoparticles, ZIF-8 and FMZ were synthesized according to the following steps: ZIF-8 was synthesized by using ion deposition. For the synthesis of ZIF*-*8*,* Zn (NO_3_)_3_·6H_2_O (10 mmol) and 2-methylimidazole (50 mmol) were dissolved in methanol (50 mL) separately. The solution containing 2-methylimidazole was added dropwise to another solution (i.e., Zn (NO_3_)_3_·6H_2_O) and reacted for 24 h at room temperature. The mixed solution was subjected to centrifugation, washed with ethanol, and drying overnight in a conventional oven.

For the synthesis of FMZ, first, FeSO_4_·7H_2_O (0.28 mmol), MnCl_2_·4H_2_O (0.28 mmol), and Zn (NO_3_)_3_·6H_2_O (2.24 mmol) were suspended into the methanol solution and stirred for 30 min. Next, the imidazole solution (30 mL) was slowly added to the aforementioned solution with stirring for 30 min. The mixture was then agitated for 12 h. The homogeneous solution was centrifuged, washed several times with methanol, and dried overnight in a vacuum oven.

### Synthesis of B@FMZ Nanoparticles

The BS NRs (1 mmol) and FMZ (10 mmol) were added to a methanol solution (50 mL), and the mixture was stirred for 24 h. Next, the resulting solution was centrifuged, rinsed with methanol, and dried in a vacuum oven for overnight. The final dried product was B@FMZ nanoparticles.

### MTX Loaded on B@FMZ Nanoparticles (B@FMZ/MTX)

B@FMZ (10 mg) and MTX (5 mg) were dissolved in ethanol and stirred overnight. The solution containing the nanoparticles was centrifuged and rinsed three times with ethanol. The samples were then dried overnight in a vacuum oven. The reaction was performed in the dark condition. The UV–vis absorbance spectrometer was used at a wavelength of 303 nm to measure the free MTX at different concentrations to obtain the standard curve of MTX. The loading efficiency of MTX in B@FMZ nanoparticles was evaluated with the standard curve of MTX. The drug loading rate was determined by using the following equation:
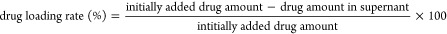


### Evaluation of Drug Release Performance

Drug release performance was investigated at different pH levels. B@FMZ/MTX nanoparticles (10 mg) were suspended in PBS at different pH values (5.5 and 7.4) and sealed in a dialysis bag. The MTX release performance was recorded by using a UV–vis spectrophotometer, and all samples were analyzed under 808 nm laser irradiation.

### Evaluation of the Photothermal Effect

The photothermal characteristics of the B@FMZ nanoparticles were examined under 808 nm laser irradiation (1 W/cm^2^, 10 min) at various concentrations. A three-cycle NIR laser on/off study was used to examine the photothermal stability, and temperature fluctuations of the nanoparticles were observed. The photothermal conversion efficiency (η) of aqueous B@FMZ nanoparticles (1 mg/mL) was exposed under an NIR laser for 600 s, and then, the laser was turned off and settled down to cool under room temperature. The value of η was evaluated as per the previous study.^41^

### Detection of ·OH Formation

B@FMZ nanoparticle’s OH· generation performance was evaluated by the reaction of MB. B@FMZ nanoparticles (1 mg/mL), H_2_O_2_ (1 mM), and MB (0.1M, 100 μL) were deposited at pH 6.5. The mixture was then incubated for 20 min at ambient temperature. The absorbance spectra were obtained at a wavelength of 664 nm using a UV–vis spectrophotometer at various laser irradiation times. TAOH was used to detect ·OH. The NaOH aqueous solution (0.2 M) was heated to a boiling temperature, and TAOH (0.74 g) was added with continuous stirring in order to obtain a transparent solution. Subsequently, the B@FMZ nanoparticles were combined with the above solution and exposed to an 808 nm laser for different durations (0, 5, 10, 15, and 20 min). The absorbance of the solution was measured at 426 nm by using a fluorescence spectrometer.

### *In Vitro* and *In Vivo* MRI and X-ray CT Imaging Studies

For the *in vitro* T_1_-weighted MR imaging, B@FMZ nanoparticles were dissolved in agarose solution (0.5%) at different concentrations. The T_1_ relaxation times of each sample were measured by using a 7T MRI device. Tumor-bearing mice were prepared and the nanoparticles were administered intratumorally to the mice. T_1_-weighted MR images were acquired before and after administration of the B@FMZ nanoparticles. Furthermore, CT imaging equipment was used to capture the *in vitro* CT signal after dissolving B@FMZ nanoparticles in 0.5% of agarose solution at various concentrations. For *in vivo* CT imaging, B@FMZ nanoparticles were injected intratumorally, and CT signals were analyzed before and after injection into tumor-bearing mice using a CT imaging system.

### Cytotoxicity Studies

The cytotoxicity was evaluated by using human liver carcinoma cancer (HepG2) cell lines. The cells (1 × 10^5^) were cultured in a standard manner in a 96-well plate for a day. Next, the cells were washed with PBS (pH 7.4), and various concentrations of the samples (B@FMZ and B@FMZ/MTX nanoparticles) were added. After that, CCK-8 (Cell Counting Kit-8) dye was added to the cells, which were then subjected to 808 nm laser irradiation for 5 min. The aforementioned laser-treated cells were then incubated at 37 °C for 2 h. Cytotoxicity was analyzed by using a CCK-8 assay. The absorbance of the cells was measured by using an ELISA plate reader (Thermo Fisher Scientific, Finland) at a fixed wavelength of 450 nm.

### Cellular Uptake of B@FMZ/MTX Nanoparticles

The cellular uptake properties of the B@FMZ/MTX nanoparticles were evaluated by using HepG2 cells via fluorescence microscopy. The cells were cultivated (3 × 10^5^) in 35 mm culture petri dishes and incubated at 37 °C for 24 h. The culture medium was changed, and the cells were washed with PBS. Then, the B@FMZ/MTX nanoparticles (100 μg/mL) was added inside the cell solution. Further, the cells were incubated for 1 and 4 h. Subsequently, the cells were rinsed and stained with DAPI (10 μM), which acts as a nuclei maker and can bind to DNA. Fluorescence microscopy was performed on the cell images.

### *In Vitro* Detection of ROS

To identify intracellular ROS, a DCFH-DA ROS probe was used, which is quickly oxidized to DCF products. Here, HepG2 cells (3 × 10^5^) were used, and cells were grown overnight in a culture dish. The B@FMZ/MTX nanoparticles were added to the cells after the medium was aspirated, and the cells were carefully washed with PBS. Next, the cells were stained with DCFH-DA (10 μM) followed by NIR laser irradiation. The cell suspension was then incubated for 15 min. Fluorescence microscopy was used to detect the ROS levels.

### Animal Experiment

BALB/c nude mice (4 weeks old) were subcutaneously injected with HepG2 (2 × 10^6^) cells for animal experiments. After the mice’s tumor volume reached 100 mm^3^, the tumor-bearing mice were separated into three groups: control, B@FMZ/MTX nanoparticles, and B@FMZ/MTX nanoparticles under 808 nm laser irradiation (0.75 W/cm^2^, 5 min). The body weights of mice and their tumor volumes were monitored during the treatment period. The following expression was used to determine, tumor volume, , here, “*L*” denotes the tumor’s length and “*W*” stands as the width of the tumor. All mice were sacrificed after 14 days of treatment. Primary organs including the kidneys, liver, spleen, heart, lungs, and tumors were collected, and soaked in formalin. The tissue slices were stained with hematoxylin and eosin (H&E) for histological analyses. All animal experiments were conducted according to the protocols and approval of the Institutional Animal Care and Use Committee of Taipei Medical University (TMU) (document number: LAC2022-0445).

### Statistical Analysis

SPSS software (version 18.0; Chicago, IL, USA) was used to perform statistical calculations for all of the experimental data. Statistical significance was set at *p* < 0.05, denoted by the notation “*”.

## Results and Discussion

### Characterizations of B@FMZ Nanoparticles

**Figure 1:**
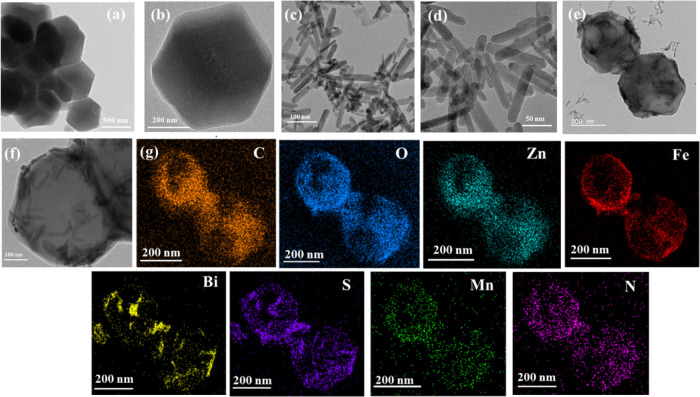
TEM images of (a, b) ZIF-8 at different magnifications, (c, d) BS NRs at various magnifications, (e, f) B@FMZ nanoparticles at different magnifications, and (g) EDS mapping of B@FMZ nanoparticles.

The TEM images of all samples are shown in Figure 1. Different magnifications of the ZIF-8 nanoparticles are listed in Figure 1a,b. ZIF-8 has a dodecahedral structure with sharp edges and a homogeneous structure. The Bi_2_S_3_ nanoparticles appeared as nanorods, and different magnifications of the BS NRs are presented in Figure 1c,d. The BS NRs exhibited a uniform structure with an average diameter of approximately 17.36 nm (Figure S1a). The BS NRs (core) were covered by Fe/Mn-doped ZIF-8 (FMZ) (shell), which can yield B@FMZ nanoparticles as a core@shell structure. The shell diameter is larger than the core, and as a result, the core@shell structure of B@FMZ nanoparticles could indeed be formed. Different magnifications of the B@FMZ nanoparticles are shown in Figure 1e,f. The B@FMZ nanoparticles have a particle size of approximately 259 nm based on dynamic light scattering (DLS) (Figure S1b). The EDS mapping is presented in Figure 1g. Additionally, the EDS element distribution presented onto the surface of B@FMZ nanoparticles is provided in Figure S1c. The above EDS results of the B@FMZ nanoparticles indicated the presence of C, N, O, Fe, Mn, Bi, and S elements, without any impurities. Therefore, the aforementioned findings indicate that the manufacture of the B@FMZ nanoparticles was successful.

**Figure 2:**
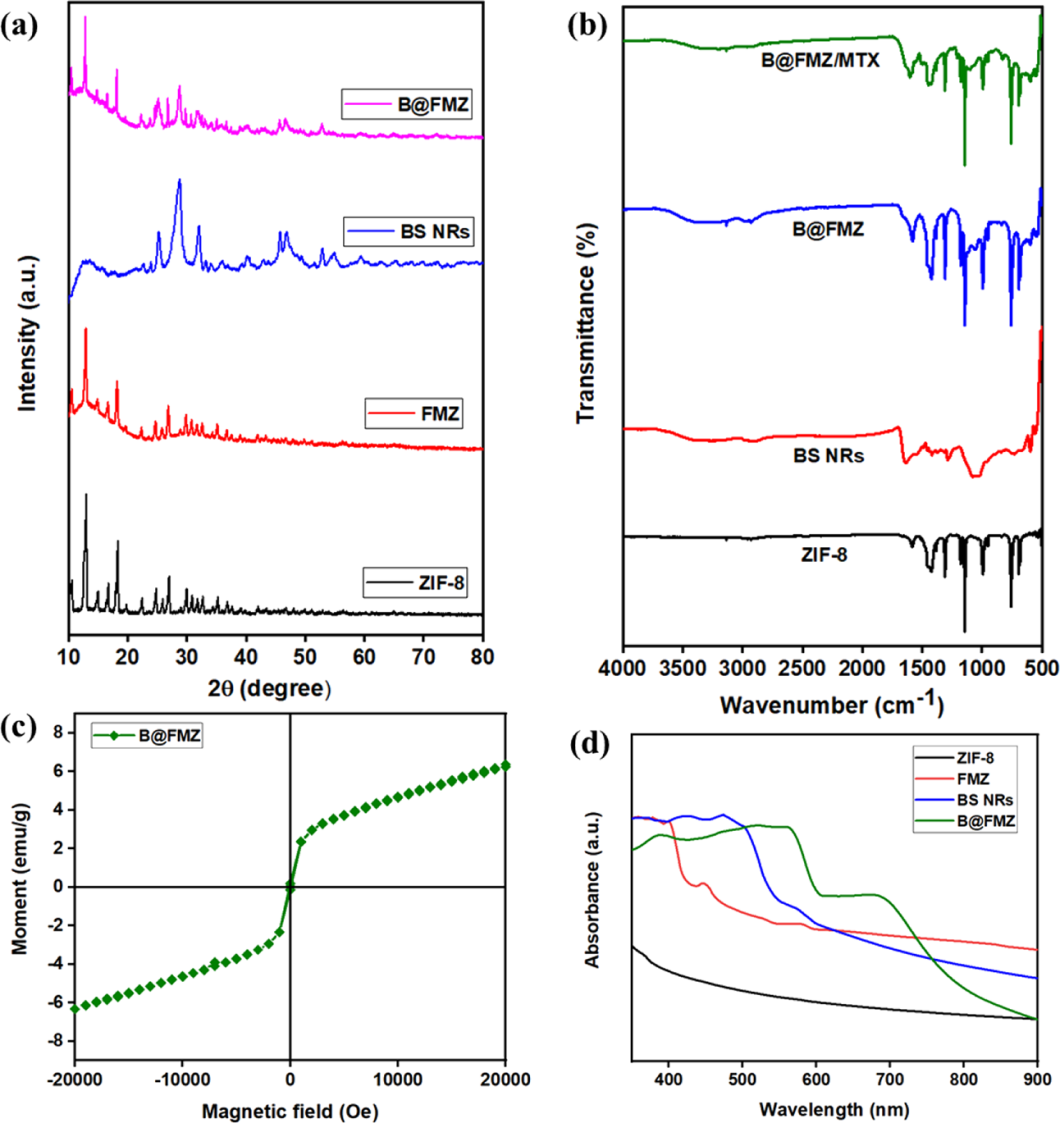
(a) XRD patterns of the ZIF-8, FMZ, BS NRs, and B@FMZ nanoparticles, (b) FTIR spectra of the ZIF-8, BS NRs, B@FMZ, and B@FMZ/MTX nanoparticles, (c) magnetic hysteresis loop of the B@FMZ nanoparticles, and (d) UV–vis–NIR absorption spectra of the ZIF-8, FMZ, BS NRs, and B@FMZ nanoparticles.

The XRD patterns of all of the synthesized samples are shown in Figure 2a. The diffraction peaks at 10.39, 12.90, 14.86, 16.55, and 18.42°, contributed to the (200), (211), (220), (310), and (222) crystal planes of ZIF-8, respectively. No additional peaks appeared for Fe/Mn-doped ZIF-8 (FMZ), suggesting that the FMZ samples did not affect the crystal structure of pure ZIF-8. The diffraction peaks at 22.70, 23.98, 25.06, 28.86, 32.02, 33.11, 45.76, 46.69, 52.86, and 54.91° corresponded to (220), (101), (130), (102), (110), (111), (200), (201), (202), and (113) crystal planes of orthorhombic Bi_2_S_3_ (JCPDS No. 23-0677). The characteristic diffraction peaks of Bi_2_S_3_ were well-indexed to the standard pattern (JCPDS No. 17-0302). Moreover, B@FMZ nanoparticles were observed in the diffraction peaks of both ZIF-8 and Bi_2_S_3_, indicating that these nanoparticles were successfully synthesized. The chemical structures of all of the samples were identified by FTIR, as shown in Figure 2b. The absorption band at 3135 and 2932 cm^–1^ was attributed to the C–H stretching vibration in imidazole and methyl group, respectively.^42^ The absorption peak at 1583 cm^–1^ corresponds to the C=N stretching vibration, and those at 1145 and 991 cm^–1^ are assigned to the C–N stretching vibrations of ZIF-8.^38^ In-plane imidazole ring bending modes are linked to the bands in the spectral area region of 950–1350 cm^–1^, whereas the bands below 800 cm^–1^ represent out-of-plane bending modes.^43^ The stretching vibration of the O–H group was assisted by the broad band at 3330 cm^–1^. The absorption bands at 1454 and 1381 cm^–1^ are attributed to the symmetric bending of the C–H group. The absorption band at 1300–1400 cm^–1^ could be assigned to the Bi–S vibration for BS NRs. A weak peak was observed at 2916 cm^–1^ owing to the C–H bond of the −CH_3_ groups.^44^ The vibrational peaks at approximately 731, 670, and 545 cm^–1^ were ascribed to the presence of Bi–S bonds, confirming the formation of BS NRs.^45^ For the B@FMZ nanoparticles, a slight shift in the peaks was observed at 3297 and 1056 cm^–1^, which was ascribed to the stretching vibration of the O–H group stretching vibration. The characteristic peaks matched well with than that of ZIF-8 and BS NRs for the B@FMZ nanoparticles. Moreover, the introduction of Fe and Mn into ZIF-8 did not alter its functional group of ZIF-8, which is consistent with the XRD results. The remaining characteristic peaks of the B@FMZ nanoparticles did not change, indicating successful fabrication. For B@FMZ/MTX nanoparticles, the absorption peaks at 2951 cm^–1^ correspond to the presence of carboxylic group, and the peaks at 1648 and 1603 cm^–1^ indicate the presence of MTX. The absorption peaks within the region of 1603 and 1543 cm^–1^ were attributed to the presence of MTX with C=C and C=O bonds, respectively. Moreover, the peaks at 1480 and 1210 cm^–1^ correspond to C–C–C and C–H bonds, respectively.^46^ In the B@FMZ/MTX nanoparticles, the distinctive peaks match well with the free MTX (Figure S2). Furthermore, results of free MTX or MTX loaded onto the B@FMZ nanoparticles are matched with the previous studies.^47,48^ As compared to B@FMZ nanoparticles, the peaks between 1400 and 1430 cm^–1^ were shifted to the higher wavenumber in the B@FMZ/MTX nanoparticles, which indicates possible interaction between MTX and nanoparticles. Therefore, the aforementioned results indicate the successful loading of MTX onto B@FMZ nanoparticles. The nitrogen adsorption/desorption of B@FMZ nanoparticles shows a type-I isotherm (Figure S3). The BET surface area and pore size of B@FMZ nanoparticles were 250 m^2^/g and 20.5 nm, repsectively. The magnetization curve of the B@FMZ nanoparticles is shown in Figure 2c. The saturation magnetization of the B@FMZ nanoparticles is 6.34 emu g^–1^. Thus, the magnetic hysteresis loop curves of these nanoparticles indicated superparamagnetic properties with intense magnetic properties for targeting tumor accumulation in biomedical applications. The UV–vis–NIR absorption spectra of ZIF-8, FMZ, BS NRs, and B@FMZ nanoparticles are shown in Figure 2d. The doping of Fe/Mn with ZIF-8 improved the absorption in the visible region for FMZ nanoparticles, indicating the enhancement of the light absorption range of ZIF-8 after the addition of Fe/Mn. In the NIR region, BS NRs exhibited a broad absorption peak. The B@FMZ nanoparticles exhibited intense NIR absorption in the range 700–900 nm, which could be used as a promising photothermal property for cancer treatment. The UV–vis–NIR absorbance spectra of B@FMZ/MTX nanoparticles are presented in Figure S4. In this Figure S4, an absorbance peak at 303 nm confirmed that the MTX was successfully loaded onto the surface of B@FMZ nanoparticles.^49^

**Figure 3:**
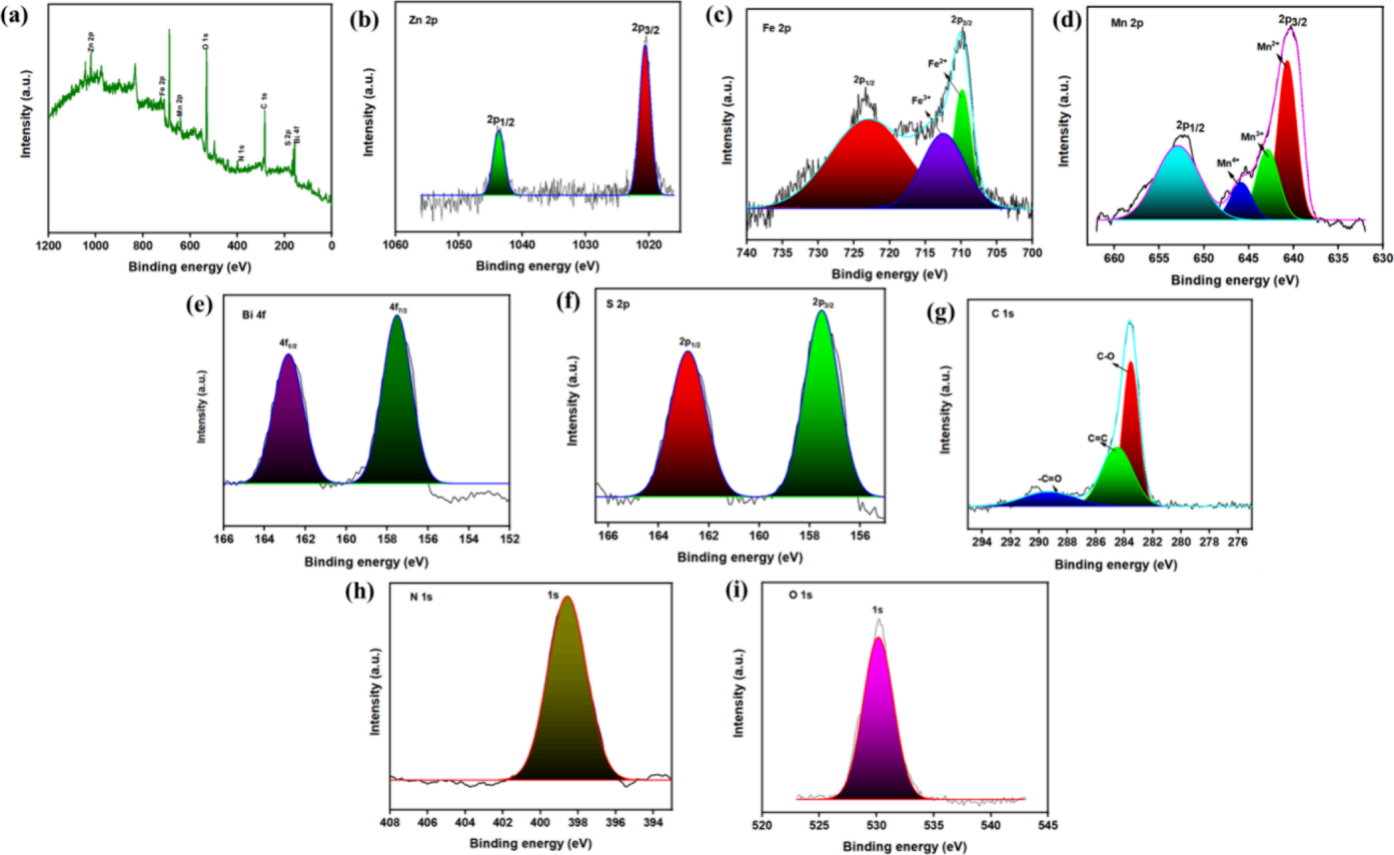
XPS spectra of the B@FMZ nanoparticles for (a) survey spectrum, (b) Zn 2p, (c) Fe 2p, (d) Mn 2p, (e) Bi 4f, (f) S 2p, (g) C 1s, (h) N 1s, and (i) O 1s.

The XPS analysis of the B@FMZ nanoparticles is shown in Figure 3. The B@FMZ nanoparticles exhibited the characteristic peaks of Zn 2p, Fe 2p, Mn 2p, Bi 4f, S 2p, C 1s, N 1s, and O 1s in the XPS survey spectrum (Figure 3a). The XPS spectra of Zn 2p showed Zn 2p_1/2_ (1043.63 eV) and Zn 2p_3/2_ (1020.50 eV) peaks, indicating the presence of Zn^2+^ (Figure 3b). The Fe 2p spectra exhibit Fe^2+^ (709.88 eV) and Fe^3+^ (712.64 eV), as shown in Figure 3c.^19^ From Figure 3d, the Mn 2p spectra showed the existence of Mn^2+^ (640.56 eV), Mn^3+^ (641.62 eV), and Mn^4+^ (643.73 eV).^2^ Two peaks in the Bi 4f spectrum at 163.81 and 157.50 eV corresponded to Bi 4f_5/2_ and Bi 4f_7/2_, respectively (Figure 3e). Two peaks in the S 2p spectra were seen at 163.25 and 157.8 eV, corresponding to S 2p_1/2_ and S 2p_3/2_, respectively (Figure 3f). The C 1s spectrum exhibited three peaks at 283.55 284.48, and 288.26 eV, which were attributed to C–O, C=C, and −C=O groups, respectively (Figure 3g). The N 1s and O 1s spectra exhibited signal peaks at 398.55 and 530.22 eV, respectively, as shown in Figure 3h,i. Thus, the results indicate successful fabrication of the B@FMZ nanoparticles.

### Photothermal, Chemodynamic, and Chemotherapy Activities

**Figure 4:**
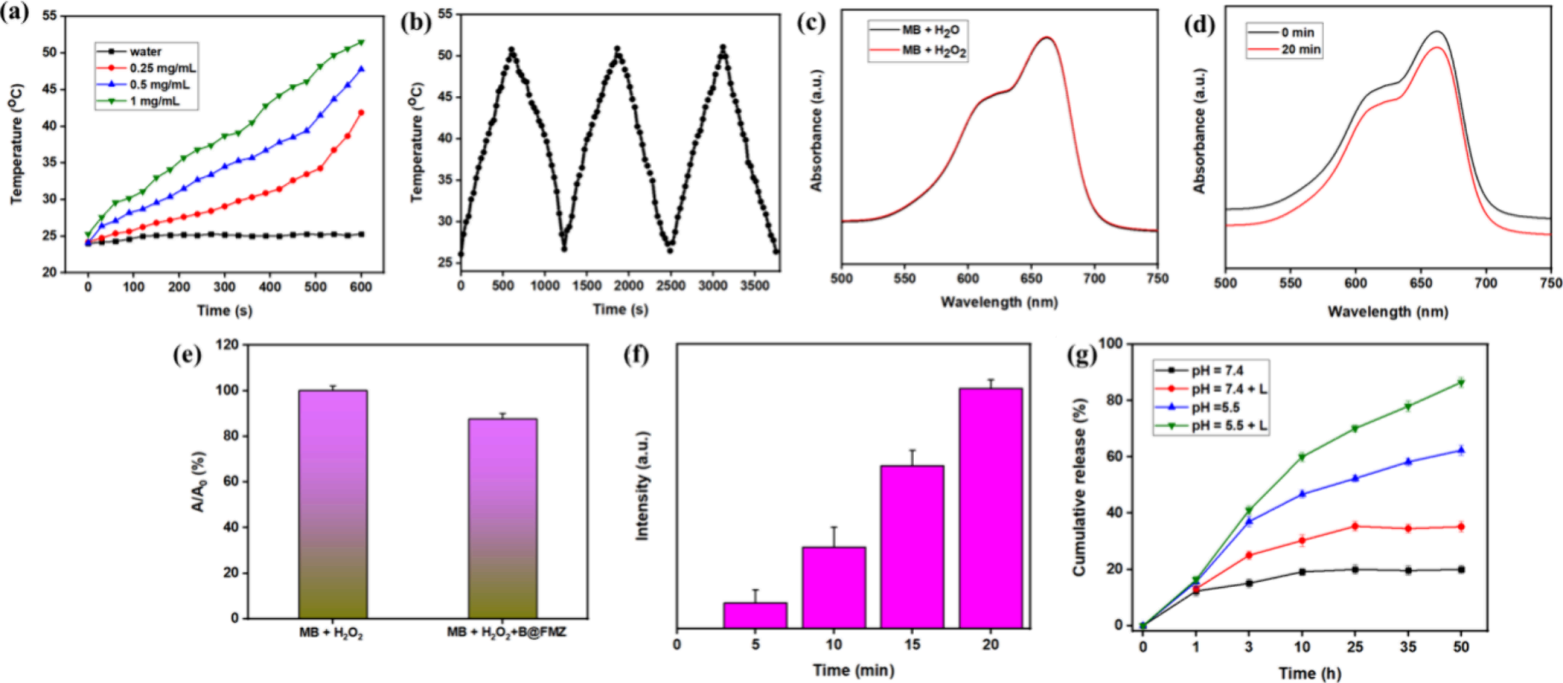
(a) Photothermal properties of the B@FMZ nanoparticles subjected to an 808 nm laser at different concentrations, (b) the photothermal stability of the B@FMZ nanoparticles exposed to 808 nm laser over three on/off cycles, (c) UV–vis spectra of the MB solution after treated with H_2_O_2_, and (d) its MB activity treated with B@FMZ nanoparticles in the presence of H_2_O_2_ at 0 and 20 min irradiation of times, (e) histogram analysis of MB degradation was monitored at 664 nm, (f) the ·OH formation was identified by TAOH in the presence of the B@FMZ nanoparticles under NIR laser irradiation, and (g) drug release performance of the B@FMZ/MTX nanoparticles at pH 5.5 and 7.4 with and without laser irradiation of 808 nm laser.

The photothermal effect of B@FMZ nanoparticles is shown in Figure 4a. The composite B@FMZ nanoparticles were irradiated at various concentrations by using an 808 nm laser. After 10 min of NIR laser irradiation, the temperature rise to 52 °C at the concentration of 1 mg/mL. On the other hand, the temperature of water was increased to only 25.3 °C. Under NIR irradiation, the temperature of the B@FMZ nanoparticles increased with nanoparticle concentration. Thus, these nanoparticles exhibited an enhanced photothermal effect, owing to their intense BS NR activity. Moreover, photothermal stability was evaluated, as shown in Figure 4b. No temperature increase was observed after three laser on/off cycles. These results indicate that B@FMZ nanoparticles exhibit excellent photothermal stability. The η value of nanoparticles was 54%, suggesting an excellent photothermal conversion performance. These findings indicate that these nanoparticles can be utilized as intense photothermal agents to inhibit cancer cells. Hence, these nanoparticles can serve as potent PTT agents for cancer treatment.

The MB degradation activity of B@FMZ nanoparticles was also assessed by ·OH. As shown in Figure 4c, the absorbance of MB in an acidic environment was not affected by H_2_O_2_. The MB absorbance intensity was reduced with an increase in the 808 nm laser irradiation in the presence of H_2_O_2_ and B@FMZ nanoparticles, which suggested that the intensity was intense ·OH generation (Figure 4d). Thus, the B@FMZ nanoparticles accelerate decomposition of H_2_O_2_. Through a Fenton-like reaction, ·OH can lead to a CDT effect. Furthermore, the photocatalytic activity of the B@FMZ nanoparticles for MB degradation was enhanced under an NIR light. Therefore, these nanoparticles can be considered to be promising CDT agents for inducing cancer cell apoptosis through NIR laser irradiation. The absorbance was decreased when it was oxidized ·OH. After incubation with H_2_O_2_ and B@FMZ nanoparticles, the absorption of MB was decreased from 100 to 87.6%, which indicated that intensive ·OH is produced via a Fenton-like reaction under laser irradiation (Figure 4e). Therefore, NIR laser irradiation would have improved the CDT effect. The fluorescence intensity of TAOH at 427 nm increased with increasing irradiation time, resulting in intense ·OH generation (Figure 4f). Thus, the B@FMZ nanoparticles produced an enhanced CDT effect under 808 nm laser irradiation. Hence, the PTT effect of these nanoparticles can improve the CDT effect by intensifying the ·OH production.

The MTX-loading efficiency of the B@FMZ/MTX nanoparticles was evaluated based on a standard curve obtained from the UV–vis spectra (Figure S5). The loading capacity is 57.49%. These results indicate that B@FMZ/MTX nanoparticles could be used as potent drug nanocarriers. The release of MTX from the nanoparticles was evaluated in a pH-dependent manner with and without laser irradiation (Figure 4g). The MTX release from B@FMZ/MTX nanoparticles at pH 5.5 was 62.34%, while at pH 7.4 was only 19.97% in 50 h. These results suggest that these nanoparticles can promote efficient drug release in acidic environments. The protonated imidazolium ions in an acidic environment interact with the anionic charge of MTX. Subsequently, the covalent bond between the metal ions and imidazolium ions is destroyed by laser irradiation in an acidic environment. Thus, MTX is released from nanoparticles by the degradation of the material structure.^50^ Furthermore, MTX exhibits higher hydrophilicity in an acidic environment than in a neutral environment. The release efficiency of B@FMZ/MTX nanoparticles under laser irradiation was 87.25% at pH 5.5 and 35.18% at pH 7.4. Thus, the aforementioned findings suggest that B@FMZ/MTX nanoparticles possess excellent drug release performance under NIR laser irradiation, because the PTT effect can lead to chemotherapy efficiency.

### Multimodal Imaging Guided of B@FMZ Nanoparticles

**Figure 5:**
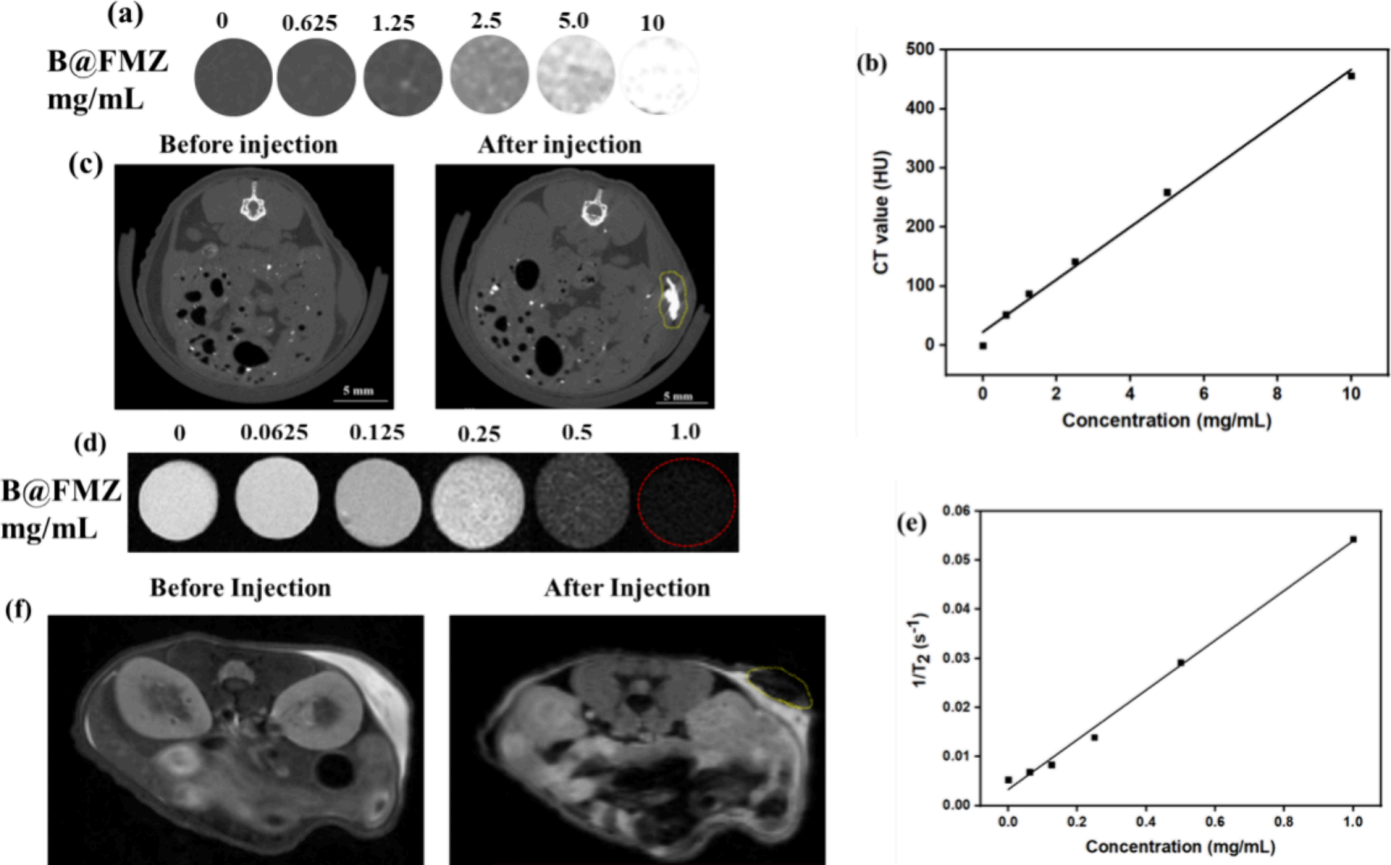
(a) CT images of the B@FMZ nanoparticles, (b) CT values of the B@FMZ nanoparticles at different concentrations, (c) CT images of tumor-bearing mice before and after injection of the B@FMZ nanoparticles, (d) MRI images of the B@FMZ nanoparticles with different concentrations, (e) T_2_-weighted relaxivity of the B@FMZ nanoparticles, and (f) T_2_-weighted MRI images of *in vivo* tumor-bearing mice acquired before and after B@FMZ nanoparticle administration.

X-ray CT images of the B@FMZ nanoparticles were evaluated at various concentrations, as shown in Figure 5. Different concentrations of B@FMZ nanoparticles were recorded by using Hounsfield (HU) values. Increasing the concentration of B@FMZ nanoparticles intensified the CT signal (Figure 5a). Moreover, the HU values of the nanoparticles were increased linearly with increasing concentration. The HU value of B@FMZ nanoparticles was 44.36 HU, which is obtained from the slope that was plotted between HU units against the concentration gradient through a linear regression diagram (Figure 5b). Therefore, the results indicated that the B@FMZ nanoparticles performed intense CT imaging owing to the presence of BS NRs as potential CT contrast agents. For *in vivo* CT imaging study, the B@FMZ nanoparticles were intratumorally injected in the tumor-bearing mice and the delivery of nanoparticles at the tumor site resulted in an intense CT signal when compared to the control group (before the injection of nanoparticles), as shown in Figure 5c. Thus, B@FMZ nanoparticles can be considered to be promising CT contrast agents for CT imaging *in vivo*. Furthermore, MR imaging of the B@FMZ nanoparticles was performed, and the results are presented in Figure 5d–f. T_2_-weighted MR imaging of the nanoparticles was performed at different concentrations. Darker T_2_-weighted MR imaging showed an increase in the nanoparticle concentration. The *r*_2_ values were obtained from the slopes plotted against concentration and relaxation rates (Figure 5e). The *r*_2_ value was 5.38 mg^–1^s^–1^. Thus, the nanoparticles are promising T_2_-weighted MR contrast agents because of the intense paramagnetic properties of the iron ions in the B@FMZ nanoparticles. *In vivo* MR imaging studies were also performed, as shown in Figure 5f. Tumor-bearing mice were intratumorally administered with B@FMZ nanoparticles. After the administration of nanoparticles into the tumor region, an intense MR signal was generated compared with that in the control group (before injection). Therefore, the aforementioned results suggested that B@FMZ nanoparticles can serve as potential MR contrast agents for cancer diagnostics.

### Cytotoxicity of B@FMZ/MTX Nanoparticles

**Figure 6:**
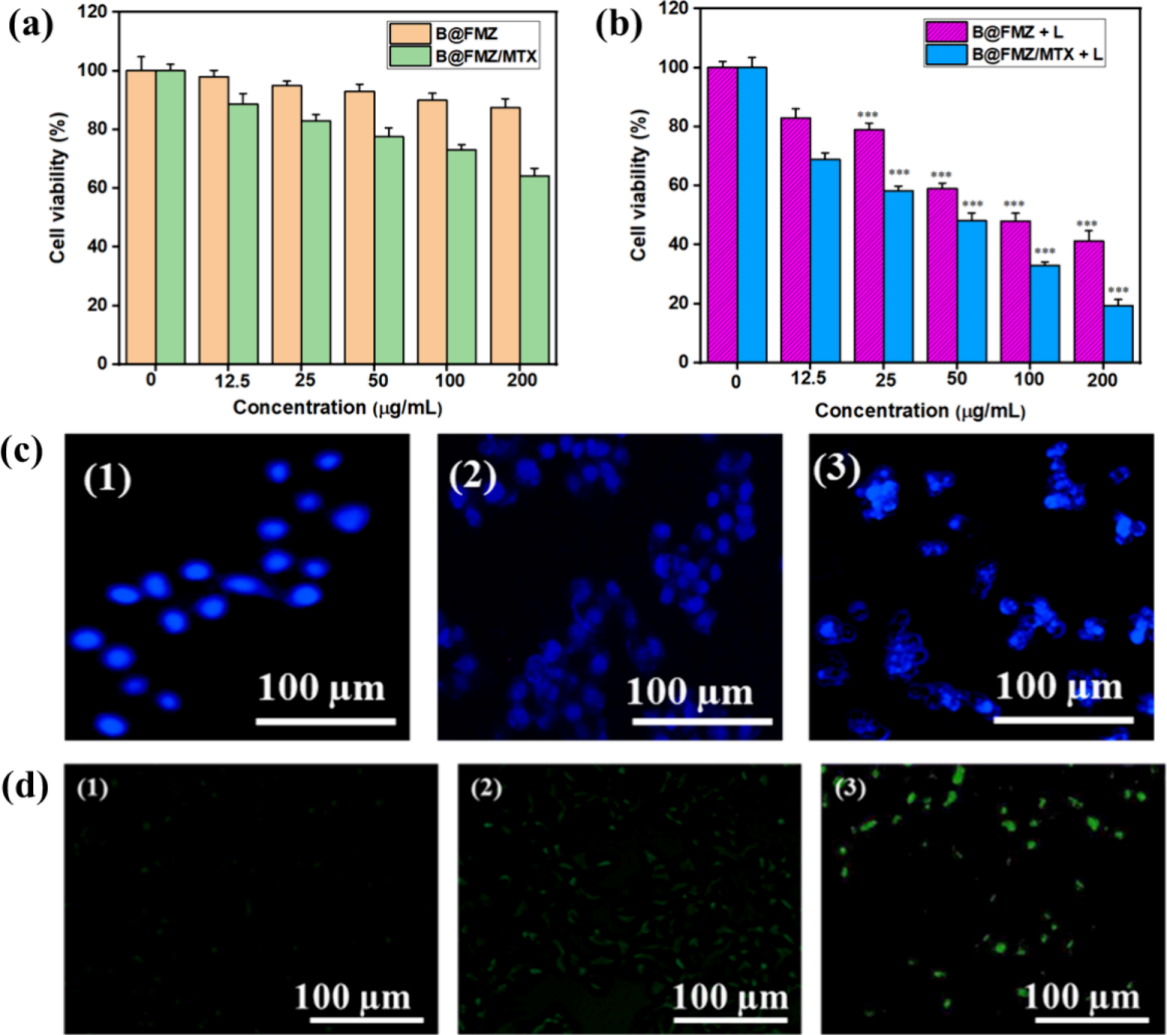
(a) Viability of human liver carcinoma cancer (HepG2) cells at different concentrations of B@FMZ and B@FMZ/MTX nanoparticles, (b) cytotoxicity of HepG2 cells treated with B@FMZ and B@FMZ/MTX nanoparticles at various concentrations with or without 808 nm laser irradiation (1 W/cm^2^, 5 min, (*** denotes *p* < 0.001), (c) HepG2 cells stained with DAPI to detect cellular apoptosis (1) control, (2) 50 μg/mL, and (3) 100 μg/mL, and (d) fluorescence images of HepG2 cells stained with DCFH-DA (1) control, (2) B@FMZ/MTX nanoparticles, and (3) B@FMZ/MTX nanoparticles were exposed under irradiation of 808 nm (scale bar = 100 μm).

The viability of HepG2 cells cultured with various concentrations of B@FMZ/MTX nanoparticles at various concentrations is shown in Figure 6a. The cell viability of B@FMZ nanoparticles was greater than 85%, even at higher concentrations. Thus, the B@FMZ nanoparticles exhibited no toxicity even at higher concentrations. The B@FMZ/MTX nanoparticles against HepG2 cells possessed cell viability of around 68% (high concentration at 200 μg/mL), which indicated that these nanoparticles could serve as intense drug carriers. In Figure 6b, B@FMZ nanoparticles with the laser irradiation group showed severe cellular cytotoxicity with the cell viability rate decreased to 41.2% and induced due to PTT and CDT effects. The HepG2 cells treated with B@FMZ/MTX nanoparticles under laser irradiation exhibited cytotoxicity higher than those treated with B@FMZ nanoparticles (Figure 6b). The cell viability of HepG2 cells treated with B@FMZ/MTX nanoparticles decreased by 19% (i.e., cellular apoptosis 81%) under an 808 nm laser owing to the synergistic effects of photothermal therapy, chemodynamic therapy, and chemotherapy. Thus, these nanoparticles possess an intense therapeutic efficiency that can induce cancer apoptosis through combined therapeutic effects for cancer treatment. The cellular uptake behavior of B@FMZ/MTX nanoparticles was investigated by using fluorescence microscopy (Figure S6). The HepG2 cells were incubated with B@FMZ/MTX nanoparticles for various durations. In Figure S6, this suggests that the variable incubation times can allow B@FMZ/MTX nanoparticles to penetrate HepG2 cells. The cellular uptake of nanoparticles in HepG2 cells is increased with increase in the incubation time period. These nanoparticles can be effectively internalized by cells through endocytosis. Therefore, these nanoparticles possess higher cellular uptake, which could be beneficial for intense therapeutic applications. The HepG2 cells were stained with DAPI that can detect cancer cell apoptosis (Figure 6c). The enhancement of B@FMZ/MTX nanoparticle concentrations showed a greater number of apoptotic bodies with a nonuniform shape of nuclear fragmentation. In contrast, the cells in the control group appeared to have a normal shape without apoptosis. Hence, these nanoparticles can promote the cellular apoptosis of HepG2 cells. *In vitro* ROS generation was examined using DCFH-DA as a fluorescent probe, which liberated the DCF products and enhanced green fluorescence emission. HepG2 cells treated with B@FMZ/MTX nanoparticles via NIR laser irradiation exhibited an intense green fluorescence signal, as shown in Figure 6d. Conversely, the B@FMZ/MTX nanoparticles exhibited weak fluorescence signals similar to those of the control groups. Thus, B@FMZ/MTX nanoparticles were enhanced by laser irradiation ·OH that promotes the CDT effect. Moreover, PTT can stimulate the CDT effect, which leads to chemical reactions and the formation of ROS radicals. Consequently, the CDT efficacy correlates with intracellular ·OH production, which signifies the laser-induced cytotoxicity of B@FMZ/MTX nanoparticles, thus facilitating the CDT effect. Hence, these nanoparticles exhibit an improved CDT effect that can inhibit cancer cells.

### Antitumor Effect of B@FMZ/MTX Nanoparticles

**Figure 7:**
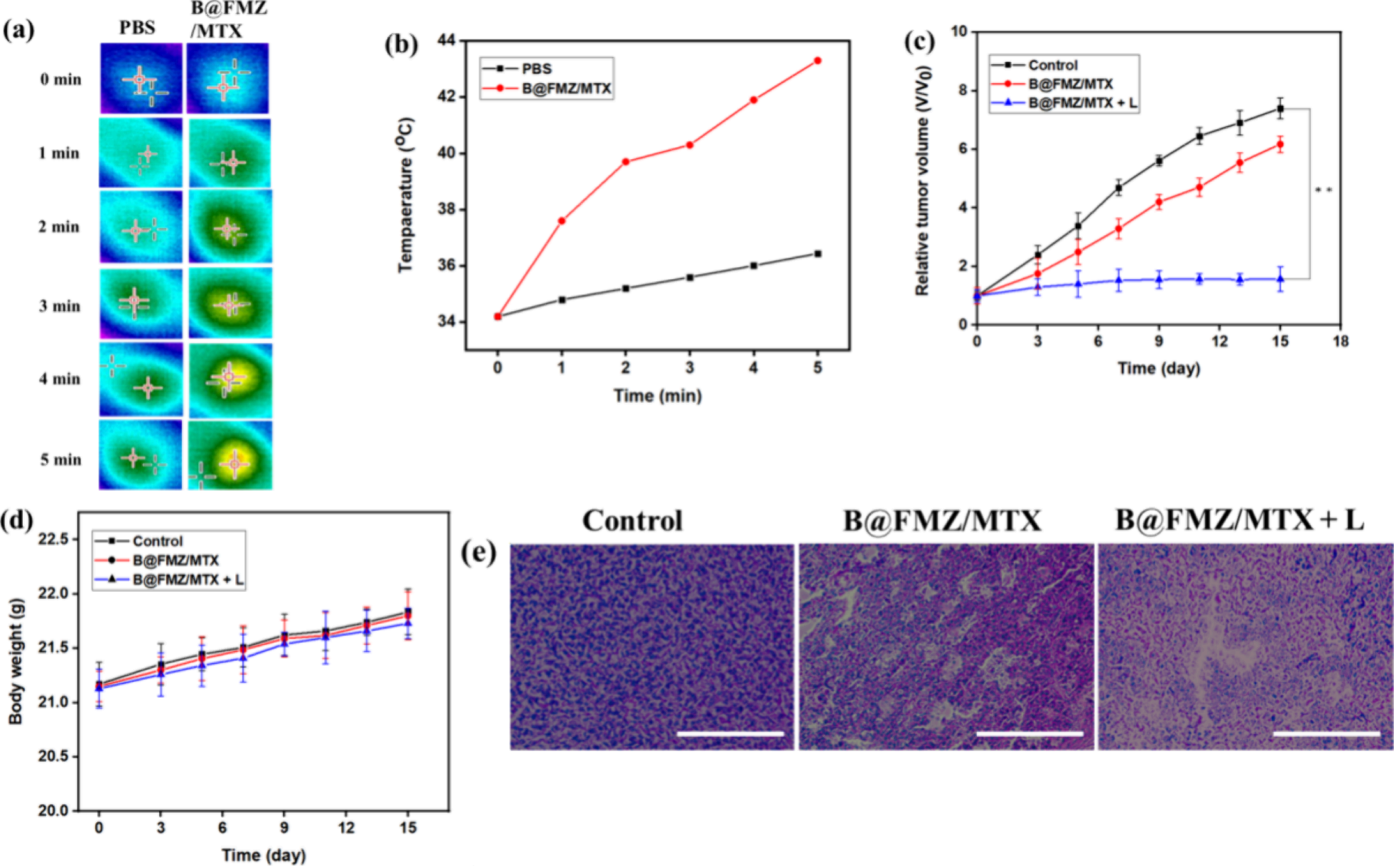
(a) Tumor-bearing mice under control and B@FMZ/MTX nanoparticles exposed to an 808 nm laser using an infrared imaging, (b) temperature change profile of tumor-bearing mice after injection of PBS and B@FMZ/MTX nanoparticles under irradiation of a NIR laser, (c) tumor volume, (d) body weight analysis of mice with various treatment groups control, B@FMZ/MTX, and B@FMZ/MTX under 808 nm laser irradiation (** denote *p* < 0.01), and (e) tumor H&E staining images after treatment groups control, B@FMZ, and B@FMZ/MTX nanoparticles with irradiation of 808 nm laser (scale bar 100 μm).

The synergistic antitumor effect of the B@FMZ/MTX nanoparticles was investigated in HepG2 tumor-bearing mice. The tumor temperatures of the mice were measured by using an infrared camera. After administration of B@FMZ/MTX nanoparticles in the tumor site, the temperature of the mice’s tumor was raised to 43.3 °C after 5 min laser irradiation, concurrently the mice injected with PBS as the control group was increased to 36.4 °C (Figure 7a,b). Thus, these results indicate that B@FMZ/MTX nanoparticles have an intense PTT effect *in vivo*, which can inhibit cancer cells. In Figure 7c, the B@FMZ/MTX nanoparticles showed better tumor growth inhibition effects primarily due to the mild CDT and chemotherapy effects. Under laser irradiation, the tumor volume of the B@FMZ/MTX nanoparticles showed a strong tumor inhibition effect owing to the synergistic effect of CDT, PTT, and chemotherapy effects. In contrast, no significant tumor ablation was observed in the control groups (Figure 7c). The body weights of the mice remained constant during the treatment period, suggesting that the B@FMZ/MTX nanoparticles had no adverse effects (Figure 7d). Thus, the B@FMZ/MTX nanoparticles exhibited enhanced antitumor effects owing to synergistic PTT, CDT, and chemotherapeutic effects when exposed to 808 nm laser irradiation. Photographic images of HepG2 tumor-bearing mice are shown in Figure S7. Figure 7e shows the H&E-stained images of the tumor tissue for each treatment group. The B@FMZ nanoparticles with the laser irradiation group exhibited a greater number of apoptotic cells than the nonirradiated group. In contrast, no changes in tumor morphology were observed in the control group. Therefore, the above results suggest that B@FMZ nanoparticles under NIR laser irradiation exhibit excellent tumor inhibition through the synergistic therapeutic effects of CDT, PTT, and chemotherapy.

**Figure 8:**
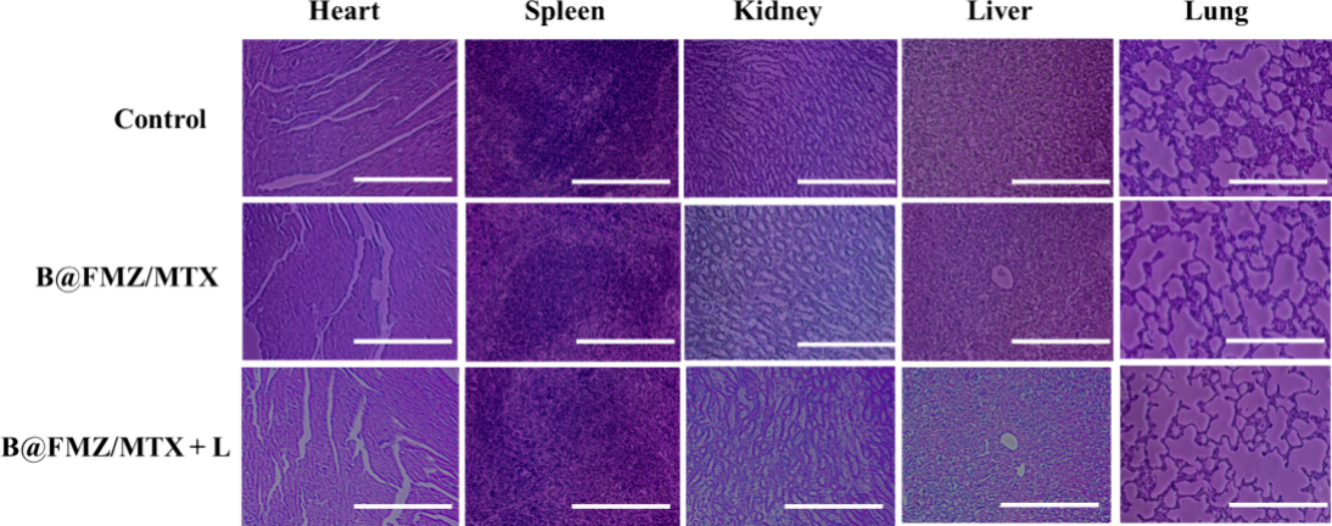
H&E-stained images of the major organs from several treatment groups: control, B@FMZ, and B@FMZ/MTX nanoparticles under 808 nm laser irradiation (scale bar 100 μm).

Figure 8 shows the major organs in the H&E-stained images. After 14 days of treatment, no aberrant inflammation was observed (Figure 8). Thus, these nanoparticles exhibit good biocompatibility and an improved antitumor efficacy through the combined therapeutic effects of CDT, PTT, and chemotherapy.

## Conclusions

In this study, B@FMZ/MTX nanoparticles were successfully fabricated. Fenton-like reactions catalyzed Fe/Mn ions through the degradation of H_2_O_2_ to produce cytotoxicity ·OH that improves the CDT effect. The B@FMZ nanoparticles exhibited a potential PTT effect owing to the presence of BS NRs and an excellent photothermal conversion efficiency of 54%. The B@FMZ nanoparticles demonstrated an intense drug-releasing efficiency of 87.25% at pH 5.5 when exposed to an NIR laser due to the PTT effect and improved the effectiveness of drug delivery. The BS NRs have high X-ray attenuation coefficients and utilized as a prominent CT imaging contrast agents. Moreover, the B@FMZ nanoparticles demonstrate superior MRI results and have the potential to be used as MRI contrast agents in cancer diagnosis. Both *in vitro* and *in vivo* findings indicated that the B@FMZ/MTX nanoparticles demonstrated improved tumor growth inhibition through the synergistic therapeutic effects of PTT, CDT, and chemotherapy. Hence, these nanoparticles have great attraction by dual-modal imaging-guided MRI/CT of intense combinational effects CDT/PTT/chemotherapy for cancer theranostics.

## Supporting Information Available

Materials and reagents; characterization; particle size of BS NRs; DLS spectrum of B@FMZ nanoparticles; EDS spectrum of B@FMZ nanoparticles; FTIR spectra of pure MTX; BET adsorption/desorption isotherm of B@FMZ nanoparticles; UV–vis–NIR absorption spectra of B@FMZ/MTX nanoparticles; the standard UV–vis absorbance curve of MTX; bright-field images of HepG2 incubated with B@FMZ/MTX nanoparticles as compared with control group for different incubation times (1 and 4 h); photograph image of HepG2 tumor-bearing mice (PDF)
