# Supplementary material for: Construction of Methotrexate-Loaded Bi2S3 Coated with Fe/Mn-Bimetallic Doped ZIF-8 Nanocomposites for Cancer Treatment Through the Synergistic Effects of Photothermal/Chemodynamic/Chemotherapy
Source: ACS Appl Mater Interfaces. 2024 Oct 17;17(1):222–34. doi: 10.1021/acsami.4c13465 (PMC11783362; doi:10.1021/acsami.4c13465)
Supplement: Supplementary file 1 — am4c13465_si_001.pdf [file am4c13465_si_001.pdf]

Supporting Information

Construction of Methotrexate Loaded Bi_2S_3 Coated with Fe/Mn-Bimetallic Doped ZIF-8 Nanocomposites for Cancer Treatment Through the Synergistic Effects of Photothermal/Chemodynamic/Chemotherapy

Pranjyan Dash [1], Nandini Nataraj [1], Pradeep Kumar Panda [2], Ching-Li Tseng [3,4,5,6], Yu-Chien Lin [1,7,8], Rajalakshmi Sakthivel [1], Ren-Jei Chung [1,9,*]

[1] Department of Chemical Engineering and Biotechnology, National Taipei University of Technology (Taipei Tech), Taipei 10608, Taiwan

[2] Department of Chemical Engineering and Materials Science, Yuan Ze University, Taoyuan City 32003, Taiwan

[3] Graduate Institute of Biomedical Materials and Tissue Engineering, College of Biomedical Engineering, Taipei Medical University, Taipei City 110, Taiwan.

[4] International Ph. D. Program in Biomedical Engineering, College of Biomedical Engineering, Taipei Medical University, Taipei city 110, Taiwan.

[5] Research Center of Biomedical Device, College of Biomedical Engineering, Taipei Medical University, Taipei city 110, Taiwan.

[6] International Ph. D. Program in Cell Therapy and Regenerative Medicine, College of Medicine, Taipei Medical University, Taipei city 110, Taiwan.

[7] School of Materials Science and Engineering, Nanyang Technological University, 50 Nanyang Avenue, Singapore 639798, Singapore

[8] ZhongSun Co., LTD, New Taipei City 220031, Taiwan

[9] High-value Biomaterials Research and Commercialization Center, National Taipei University of Technology (Taipei Tech), Taipei 10608, Taiwan

* Corresponding Author: Professor Ren-Jei Chung

Email: rjchung@ntut.edu.tw; Tel: (886-2) 2771-2171 ext. 2547

Address: Department of Chemical Engineering and Biotechnology, National Taipei University of Technology (Taipei Tech), No. 1, Sec. 3, Zhongxiao E. Rd., Taipei 10608 Taiwan

Materials and reagents

Materials

Bismuth (III) nitrate pentahydrate (Bi $(NO_3)_3.5H_2O$), polyvinylpyrrolidone (PVP), sodium sulfide nonahydrate (Na_2S. $9H_2O$) were brought from Alfa-Aser (USA). Zinc nitrate hexahydrate (Zn $(NO_3)_3.6H_2O$), 2-methylimidazole, Ferrous sulfate heptahydrate ($FeSO_4.7H_2O$), Manganese (II) chloride tetrahydrate ($MnCl_2.4H_2O$) were purchased from Sigma-Aldrich (USA). Sodium hydroxide (NaOH), and Terephthalic acid (TAOH) were bought from Sigma-Aldrich (USA). Methotrexate (MTX) was bought from United states pharmacopeia (USP) reference standard, acetic acid (99.8%), sulfuric acid (98%), methyl hydroxide (MeOH), ammonium hydroxide (NH_4OH) and ethanol (99.8%) were purchased from Honeywell (USA). Other chemicals were purchased from a registered vendor and used directly.

Characterization

The nanoparticles chemical analysis studies were using Fourier transform infrared (FTIR) spectrometer (PerkinElmer Spectrum 65, USA). The nanoparticles surface morphologies and

their elemental compositions studies were evaluated using Transmission electron microscopy (TEM) (JEM2100F, JEOL, Japan) and energy-dispersive electron microscopy (EDS) respectively. The materials crystal structure was investigated using X-ray diffraction (XRD) (X' pert 3Powder, Malvern, PANalytical, Netherlands). The optical properties of samples were investigated using UV-Vis spectroscopy (JASCO 650, Japan). X-ray photoelectron spectroscopy (XPS, JPS-9030, JEOL, Japan) was employed using an ESCALAB250 system coupled with an Al K_{α} X-ray source. Brunauer–Emmett–Teller (BET) was investigated the surface area of samples (Micromeritics, Gemini V, Germany).

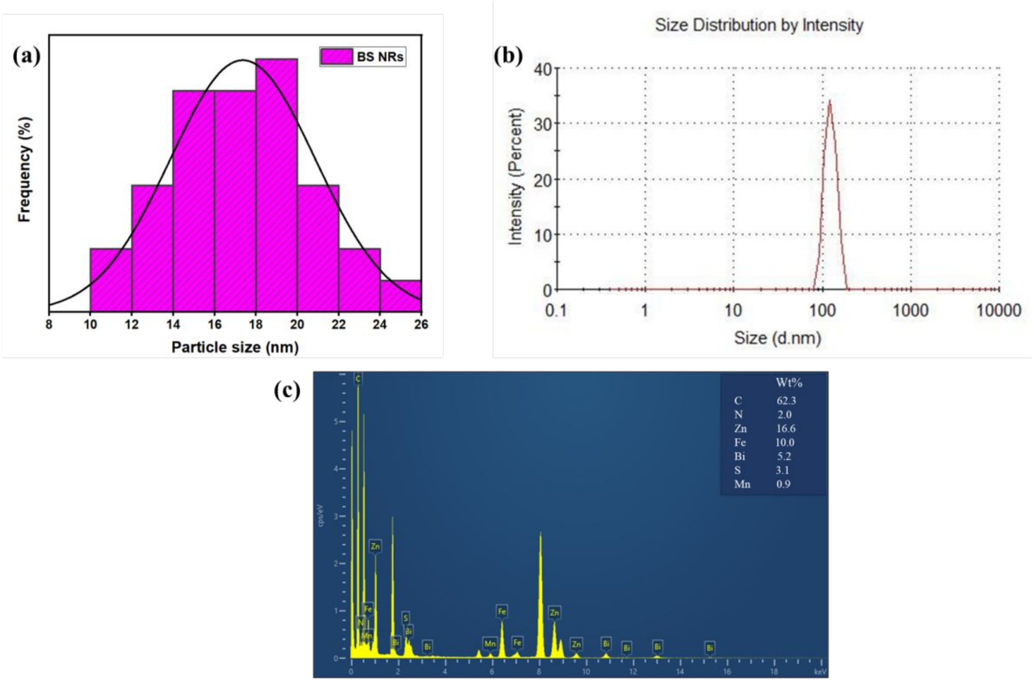

Figure S1. (a) Particle size of BS NRs, (b) dynamic light scattering (DLS) spectrum of B@FMZ nanoparticles and (c) EDS spectrum of B@FMZ nanoparticles.

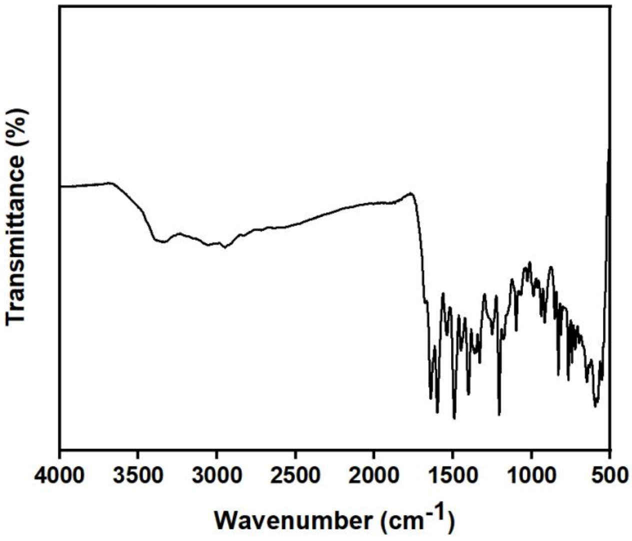

Figure S2. FTIR spectra of free MTX.

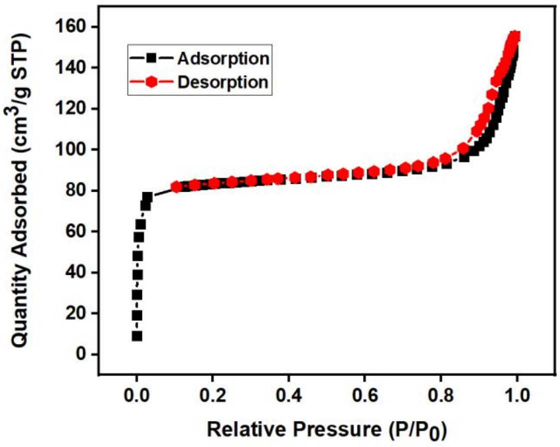

Figure S3. The N_2 adsorption/desorption isotherm of B@FMZ nanoparticles.

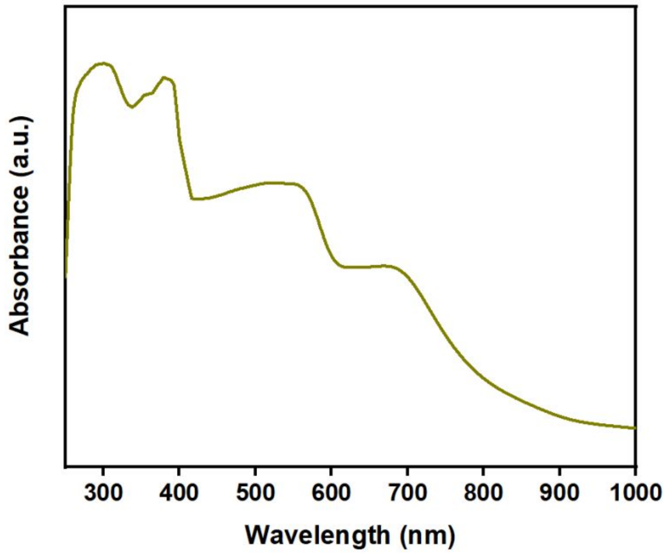

Figure S4. UV-Vis-NIR absorption spectra of B@FMZ/MTX nanoparticles.

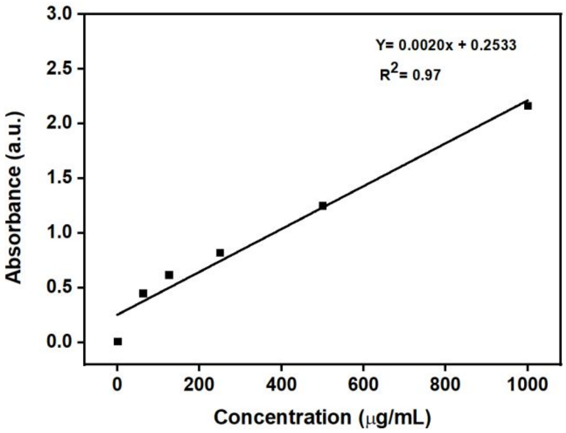

Figure S5. The standard UV-vis absorbance curve of MTX.

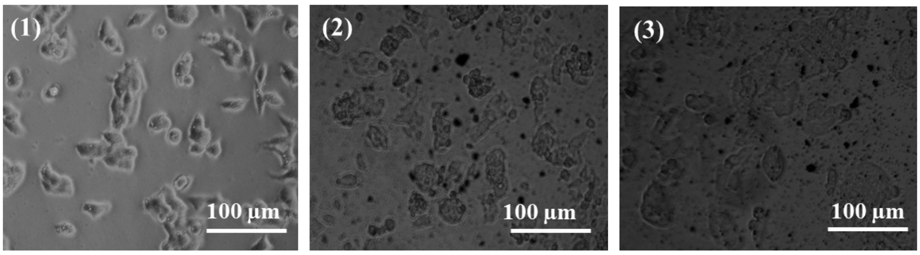

Figure S6. Bright-field images of HepG2 incubated with B@FMZ/MTX nanoparticles for different incubation times (1) control, (2) 1 h, and (3) 4 h.

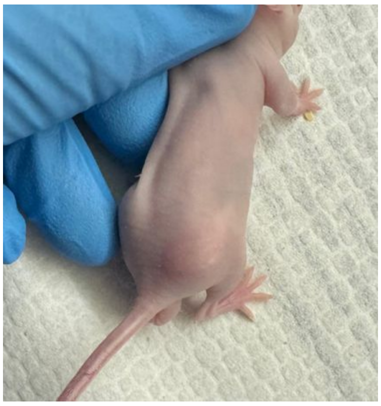

Figure S7. Photograph image of HepG2 tumor bearing mice.
